# Inhibition of herpes simplex virus-1 infection by MBZM-N-IBT: in silico and in vitro studies

**DOI:** 10.1186/s12985-021-01581-5

**Published:** 2021-05-26

**Authors:** Abhishek Kumar, Saikat De, Alok Kumar Moharana, Tapas Kumar Nayak, Tanuja Saswat, Ankita Datey, Prabhudutta Mamidi, Priyadarsee Mishra, Bharat Bhusan Subudhi, Soma Chattopadhyay

**Affiliations:** 1 Institute of Life Sciences, Autonomous Institute of Dept of Biotechnology (Govt of India), Nalco Square, Bhubaneswar, 751023 India; 2 School of Pharmaceutical Sciences, Siksha O Anusandhan Deemed To Be University, Khandagiri Square, Bhubaneswar, 751023 India

**Keywords:** Herpes simplex virus-1, MBZM-N-IBT, ICP8, gC, UL9

## Abstract

**Introduction:**

The emergence of drug resistance and cross-resistance to existing drugs has warranted the development of new antivirals for Herpes simplex viruses (HSV). Hence, we have designed this study to evaluate the anti-viral activity of 1-[(2-methyl benzimidazole-1-yl) methyl]-2-oxo-indolin-3-ylidene] amino] thiourea (MBZM-N-IBT), against HSV-1.

**Method:**

Molecular docking was performed to assess the affinity of MBZM-N-IBT for HSV-1 targets. This was validated by plaque assay, estimation of RNA and protein levels as well as time of addition experiments in vitro.

**Result:**

Molecular docking analysis suggested the inhibitory capacity of MBZM-N-IBT against HSV-1. This was supported by the abrogation of the HSV-1 infectious viral particle formation with the IC_50_ value of 3.619 µM. Viral mRNA levels were also reduced by 72% and 84% for UL9 and gC respectively. MBZM-N-IBT also reduced the protein synthesis for gC and ICP8 significantly. While mRNA of ICP8 was not significantly affected, its protein synthesis was reduced by 47%. The time of addition experiment revealed the capacity of MBZM-N-IBT to inhibit HSV-1 at early as well as late stages of infection in the Vero cells. Similar effect of MBZM-N-IBT was also noticed in the Raw 264.7 and BHK 21 cells after HSV-1 infection. Supported by the in silico data, this can be attributed to possible interference with multiple HSV targets including the ICP8, ICP27, UL42, UL25, UL15 and gB proteins.

**Conclusion:**

These results along with the lack of acute oral toxicity and significant anti-inflammatory effects suggest its suitability for further evaluation as a non-nucleoside inhibitor of HSV.

**Supplementary Information:**

The online version contains supplementary material available at 10.1186/s12985-021-01581-5.

## Introduction

Herpes simplex viruses (HSV) are double-stranded DNA virus of *Herpesviridae* family. Infections due to Herpes simplex virus type 1 and 2 (HSV-1 and HSV-2) are very common in the world, where about 67% of the population is suffering from it (http://www.who.int/mediacentre/factsheets/fs400/en/). Following infection, life-long latency is usually established leading to recurrence of the infections [1]. Although in many cases the infections are not life-threatening, serious illness can be induced in the immunocompromised population [2]. It is also known to cause infection in central nervous system leading to serious consequences [3].

Infections due to Herpes Simplex Virus (HSV) are primarily managed by treatment with nucleoside derivatives including acyclovir, ganciclovir, penciclovir, valaciclovir, and famciclovir [4]. However, the emergence of drug resistance has posed a serious challenge to their efficacy. The mechanism of resistance is generally attributed to either mutation in HSV thymidine kinase (TK) or DNA polymerase [5]. Since other drugs of this class also share a similar mode of action, cross-resistance is reported among drug-resistant strains [6]. As an alternative strategy, efforts have been made to develop non-nucleoside inhibitors of HSV. A human apolipoprotein E mimetic dimer peptide has been shown to inhibit HSV-1 in a mouse ocular model following topical application [7] Foscarnet, a structural analogue of the anion pyrophosphate blocks the pyrophosphate binding on viral DNA polymerases to inhibit HSV. As it is not dependent on viral protein kinases, it can be considered useful against acyclovir-resistant HSV. However, emergence of resistance to foscarnet [8, 9] has encouraged further exploration of new non-nucleoside inhibitors against HSV.

Thiosemicarbazone derivatives have long been associated with antiviral properties [10]. They are also widely reported for antiviral effects against HSV [11]. Although the mode of antiviral action of most of these compounds is not clear, few including 5-amino-1-formyl-4-methylisoquinoline thiosemicarbazone (MAIQ) has been reported to reduce HSV-1 replication by inhibition of viral Ribo Nucleotide Reductase (RNR) [12]. However, considering their wide antiviral properties, other modes of antiviral action cannot be ruled out. In spite of the limited clinical success so far, thiosemicarbazone remains one of the important group that attracts attention for the development of antiviral compounds. Several optimizations have been adopted to improve its therapeutic efficacy. Recently, we developed MBZM-N-IBT as a hybrid of isatin-β-thiosemicarbazome (IBT) and 2-methyl benzimidazole [13]. This showed significant inhibition of Chikungunya virus (CHIKV) replication unlike the prototype of IBT (Methisazone). It also showed interferences in various stages of CHIKV infection with a good selectivity index (CC50/IC50 =  > 21). Its antiviral property against CHIKV might not ensure its antiviral action against HSV. However, methisazone was shown to have effect against HSV infection [14]. Considering the significantly higher activity of MBZM-N-IBT against CHIKV as compared to methisazone [13], it would be interesting to see if it also has anti-HSV activity. With these considerations and the need to find new hit against HSV, the potential of MBZM-N-IBT against HSV-1 was evaluated in this study.

## Materials and methods

### Cells, virus, antibodies and drugs

The Vero cells (African green monkey kidney epithelial cells) and the BHK 21 cells (Baby hamster Kidney epithelial cells) were maintained in the Dulbecco’s modified Eagle’s medium (DMEM; PAN Biotech, Aidenbach, Germany) supplemented with 5% Fetal Bovine Serum (FBS, PAN Biotech, Aidenbach, Germany), 0.1% Gentamycin and 1% Penicillin–Streptomycin (PAN Biotech, Aidenbach, Germany). The Raw264.7 cells (mouse monocyte/macrophage cell line), was maintained in RPMI-1640 (Himedia Laboratories Pvt. Ltd, Mumbai, India) supplemented with 2.0 mM L-glutamine, Penicillin 100 U/mL, Streptomycin 0.1 mg/mL and 10% Fetal bovine serum (FBS; PAN Biotech, Germany) at 37 °C under a humidified incubator with 5% CO_2_. The HSV-1 virus strain KOS with GenBank accession Number JQ673480.1 [15] was kindly gifted by Dr. Roger Everett, Glasgow University, Scotland. MBZM-N-IBT was synthesized by our group [13] and acyclovir was procured from Sigma (Sigma, USA). The anti-ICP8 (ab20194), anti-gC (ab6509) monoclonal antibodies and GAPDH antibody were procured from Abcam (Cambridge, UK) and Abgenex Pvt. Ltd. (Bhubaneswar, India) respectively.

### Molecular docking

The molecular docking of MBZM-N-IBT was carried out following the method reported previously [13, 16]. In brief, the target proteins involved in entry, replication, packaging, and release of HSV for which well-resolved structures are available, were recovered from the protein data bank. The structures of the proteins were optimized by extraction of any co-crystallized ligand and water molecules from the catalytic site using the Discovery studio visualize package software (Discovery studio 3.5). The ligand geometry was optimized using the Argus Lab package software (Argus Lab 4.0.1) and docked to the macromolecules using the AutoDockVina software [13, 16]. The interaction between ligand and macromolecule was visualized using the PyMOL molecular graphics system (PyMOL 1.3). The structure of HSV thymidine kinase (PDB ID: 1KI3), co-crystallised with penciclovir was used as a control. Penciclovir was removed from the crystal structure and docked using the AutoDockVina software. The most stable binding mode was compared with the experimentally reported mode of interaction to verify the reproducibility. The direct inhibitor of the target molecule (wherever available), was taken as a standard for the comparison of the binding affinity.

### HSV-1 infection

One day before infection, the Vero/BHK 21/Raw 264.7 cells were seeded into 6-well cell culture dishes (TPP, Trasachingen, Switzerland). Next day, HSV-1 infection was carried out in the 100% confluent cells with multiplicity of infection (MOI) 1 as described earlier [17]. The infected cells were examined under a microscope (Magnification 20X) and pictures were taken at 24 h post infection (hpi) for the detection of cytopathic effect (CPE).

### RNA extraction and RT-PCR

To quantitate the viral RNA, the Vero/BHK/Raw 264.7 cells were infected with HSV-1 (MOI 1) and treated with drugs. The cells were harvested after 24hpi and the total RNA was extracted from the cells using Trizol (Invitrogen, Gaithersburg, MD, USA). The cDNA was generated by the first strand cDNA synthesis kit (Fermentas, Vilnius, Lithuania) according to the manufacturer’s protocol and the viral genes (UL9, ICP8, gC and gD) were amplified along with GAPDH as a control, by using primers mentioned in the Supplemental Table 1. Intensities of all the bands were measured by the Image J (NIH, Bethesda, MD, USA) software.

**Table 1:** Binding affinities (Kcal/mol) of MBZM-N-IBT against HSV target structures

PDB ID	Targets	Binding affinities
1KI3	Thymidine kinase of HSV1	− 5.2
3F0T	Thymidine kinase of HSV1	− 4.9
1AT3	HSV-2 protease	− 5.2
1NO7	Major capsid protein of HSV1	+ 1.6
1URJ	Single stranded DNA-binding protein (ICP8) from HSV1	− 9.8
5BQK	C-terminal domain of ICP27 protein from HSV-1	− 8.2
2F5U	UL25 DNA Packaging Protein from HSV1	− 8.7
2GUM	Extracellular domain of glycoprotein B from HSV1	− 8.0
2GV9	DNA polymerase of HSV1 (UL42)	− 7.9
4IOX	DNA-packaging motor pUL15 C-terminal nuclease domain of HSV-1	− 8.3
3U82	Envelope glycoprotein D of HSV-1	− 6.8

### Plaque assay

The quantitation of the infectious virus particle release was performed by plaque assay as mentioned earlier [18]. In brief, the supernatants and the cells of the infected and MBZM-N-IBT or acyclovir treated were collected at 24 hpi. The 100% confluent cells were infected with serially diluted samples and plaques were visualized after 3–4 days. The plaque number was counted for each sample as Plaque Forming Unit/mL (PFU/ mL) and the bar diagram was generated by using the Prism software. The plaques were counted from two dishes for each three independent experiments and the data represent the mean of each independent experiments (in duplicate) ± standard deviation (SD).

### Western blot

Viral Protein expression in host cells was examined by the Western blot analysis according to the procedure described earlier [17]. Cells were harvested at different hours post infection (hpi) for ICP8 and gC and then lysed with RIPA buffer containing 300 mM NaCl, 1% NP-40, 0.1% SDS, 0.5% Sodium deoxycholate and 50 mMTris (pH 8.0). Equal amount of proteins (60 µg) were separated on a 10% SDS–polyacrylamide gel and were transferred onto the PVDF membrane. The viral proteins were detected with anti-ICP8 mAb, anti-gC mAb and GAPDH was used as a loading control. The intensities of all the individual bands were quantified by using the Image J software.

### Immunofluorescence assay

The Vero cells were grown on glass coverslips and infected as described above. At 18 hpi, coverslips were processed for confocal microscopy as mentioned previously [18]. In brief, coverslips were washed twice with cold PBS. The cells were fixed with freshly prepared 4% paraformaldehyde in PBS (pH 7.4), for 30 min at RT. Next, the cover slips were permeabilized with 0.5% Triton X100 in PBS for 5 min. After three washes, cells were blocked with 3% Bovine serum albumin (BSA; Sigma) with 10 mM glycine (Blocking solution, Sigma) in PBS overnight at RT. Cells were incubated with anti-ICP8 mAb for 1 h at RT. After washing, cells were incubated with the AF 488 conjugated anti-mouse antibody (1:15,000) for 45 min. Then, coverslips were mounted with antifade (Invitrogen) to reduce photo-bleaching. Fluorescence microscopic images were acquired using the Leica TCS SP5 confocal microscope (Leica Microsystems, Heidelberg, Germany) with 20X objectives and analyzed using the Leica Application Suite Advanced Fluorescence (LASAF) V.1.8.1 software.

### Flowcytometry (FACS)

For flow cytometric detection of the HSV-1 antigen (ICP8), cells were processed and acquired as described before [19]. Briefly, the virus infected and mock Vero cells with or without drug were harvested at 15 hpi, fixed in 4% paraformaldehyde for 10 min at RT and were washed twice in ice cold 1X PBS to remove excess paraformaldehyde. Then the cells were re-suspended in FACS buffer (1X PBS, 1% BSA, 0.01% NaN_3_) and stored at 4 °C until staining. For intra cellular staining (ICS) of HSV-1 antigen (ICP8), the cells were permeabilized in permeabilization buffer (1X PBS + 0.5% BSA + 0.1% Saponin + 0.01% NaN_3_) followed by blocking in 1% BSA (in permeabilization buffer) for 30 min at room temperature. After washing with permeabilization buffer, cells were incubated for 30 min with mouse anti-ICP8 primary antibody. Then the cells were washed 3 times with the same buffer to remove unbound primary antibodies, followed by incubation in the anti-mouse Alexa Fluor® 488 conjugated IgG (H + L) secondary antibodies. The normal mouse IgG was taken as isotype control during ICS. After staining the cells were re-suspended in the FACS buffer containing 1% paraformaldehyde (w/v) and stored at 4 °C until sample acquisition. Then, the cells were acquired by the BD FACS Calibur**™** flow cytometer (BD Biosciences, CA, USA) and analyzed by the CellQuest Pro software (BD Biosciences, CA, USA). A total of approximately ten thousand cells were acquired per sample. For analysis, dot plot quadrants were set on the basis of isotype control.

### Time of addition

After infecting the cells with HSV-1 (MOI 0.001, 2 and 5), MBZM-N-IBT (200 µM) was added to the cells at 0, 4, 8, 12, 16, 20 hpi and both the cells as well as supernatants were collected at 24 hpi. Virus titer was determined by the plaque assay according to the previously mentioned procedure.

### Statistical Analysis

The statistical analyses were performed by using the One-way ANOVA (nonparametric, and the Dunnett’s multiple comparisons test) in the Graph Pad Prism 5.0 software. The statistical analysis of the experimental data was presented as the mean ± SD of three independent experiments (n ≥ 3). The *p-value*, less than 0.001 was considered to be statistically significant.

## Results

### The affinity of MBZM-N-IBT for multiple drug targets of HSV-1

The crystal structure of HSV thymidine kinase (PDB ID: 1KI3) co-crystallized with penciclovir was used in molecular docking as a control. The binding residues of the most stable complex obtained by molecular docking were compared with that of the experimentally found binding pose. The active site residues in the experimentally resolved structure including Arg-176, Tyr-172, Gln-125, His-58 and Glu-83 were also found to interact with penciclovir in a similar manner in the predicted pose (Additional file 1: Fig. 1). Since co-crystallised ligands were not available in other target structures, it was not possible to employ separate control for each target to compare molecular docking. Hence, the binding affinity (-7.3 kcal/Mol) of HSV thymidine kinase (PDB ID: 1KI3) was taken as the cut-off to filter the HSV targets of MBZM-N-IBT (Table 1).

Higher affinities were found against target proteins with known role in HSV entry, replication, packaging and release. This encouraged further screening to find its efficacy against HSV.

### Inhibition of HSV-1 infection by MBZM-N-IBT

**Fig. 1:**
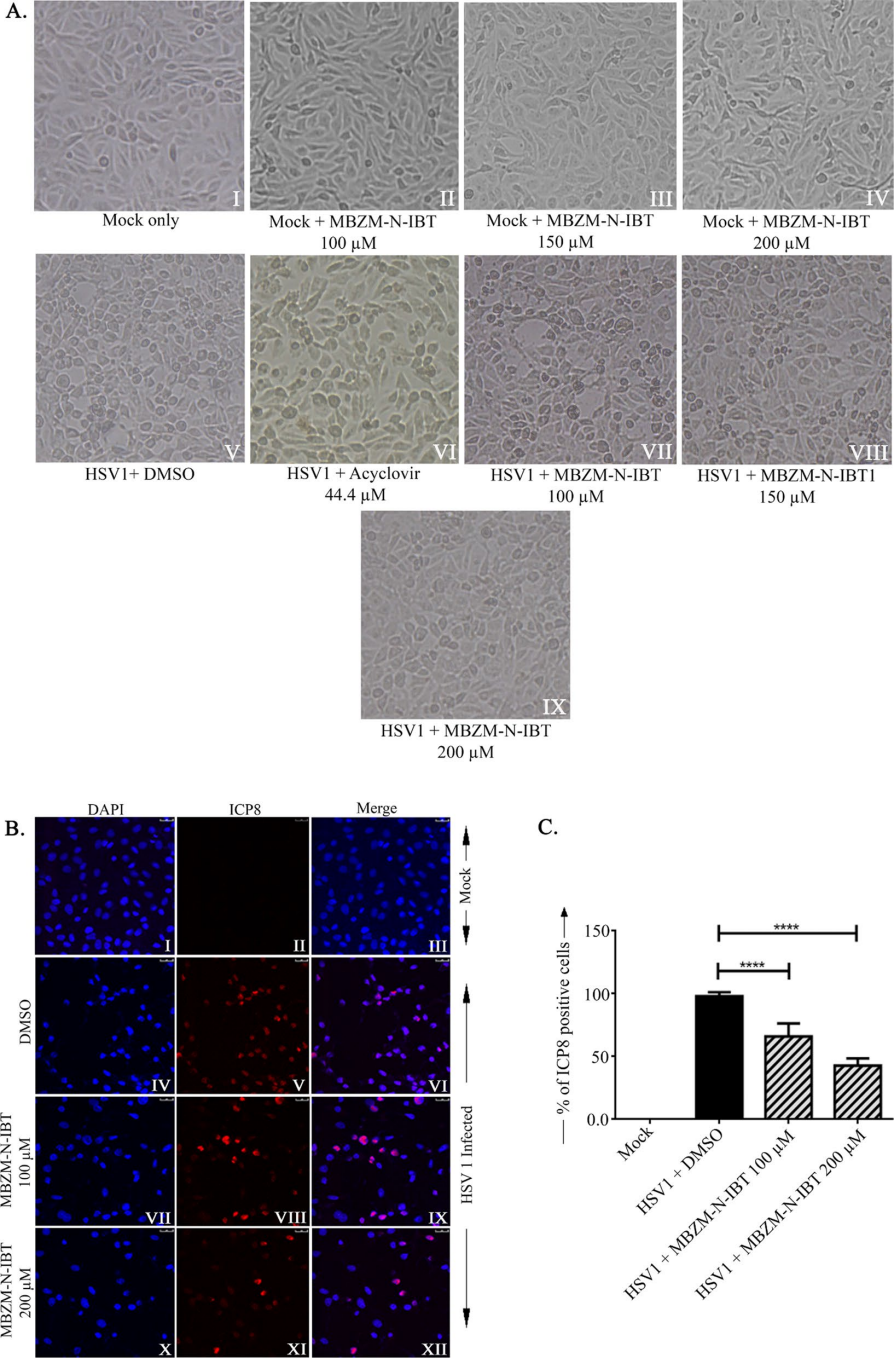
Inhibition of HSV-1 infection by MBZM-N-IBT. Vero cells were infected by HSV-1 with MOI 0.001. DMSO, 44.4 µM of Acyclovir and 100 µM, 150 µM and 200 µM of MBZM-N-IBT were added to the Vero cells. DMSO and Acyclovir were used as negative and positive controls respectively. **a** Morphological changes were observed under a microscope at 20X resolution **b **Vero cells were seeded onto cover-slips and infected with HSV-1 at MOI 1 whereas cells without HSV-1 infection were considered as Mock.. After infection, cells were treated with DMSO, 100 µM and 200 µM of MBZM-N-IBT The cells were fixed with 4% paraformaldehyde after 18 hpi and probed with ICP8 (II, V, VIII, XI) followed by staining with secondary antibody, anti-mouse Alexa Fluor 488(red) respectively. Nuclei were counterstained with DAPI (blue). **c** Bar diagrams showing the percentage of HSV-1 ICP8 positive cells in infected and drug treated samples. Data represented as mean ± SEM from three independent experiments using the one way Anova, Dunnett’s multiple comparisons test *p* ≤ 0.05 was considered statistically significant

The cytotoxic effect of MBZM-N-IBT (50 to 800 µM) on Vero cells was tested previously and the CC50 value was considered to be > 800 µM [13]. To assess the antiviral property of MBZM-N-IBT against HSV-1, both infected and uninfected Vero cells were treated with 100 µM, 150 µM and 200 µM concentrations of MBZM-N-IBT for 24 h. Acyclovir (44.4 µM) was used as a positive control [20]. No morphological change was observed in the uninfected Vero cells with or without treatment of drugs (Fig. 1a, I-IV), whereas, morphological changes were observed with HSV-1 infection (Fig. 1a, V and VI). This was reduced with increasing concentrations of MBZM-N-IBT (Fig. 1a, II-IX) indicating the inhibitory effect of this molecule for HSV-1.

In order to understand the effect of MBZM-N-IBT in HSV-1 infection, Vero cells were infected with HSV-1 and viral antigen, ICP8 was observed to be reduced significantly in confocal microscopy (Fig. 1b, c). In order to assess the inhibitory effect of MBZM-N-IBT in other cells, the BHK 21 and Raw 267.4 cell lines were infected with HSV-1 and similar level of viral inhibition was observed by plaque assay and RT-PCR (Additional file 1: Fig. 6). The cytotoxicity assay did not show any toxic effect of MBZM-N-IBT on to the BHK 21 and Raw 267.4 cells. Hence, the data suggest that MBZM-N-IBT can inhibit HSV-1 infection significantly in vitro.

### IC50 value of MBZM-N-IBT for HSV-1 is 3.619 µM

**Fig. 2:**
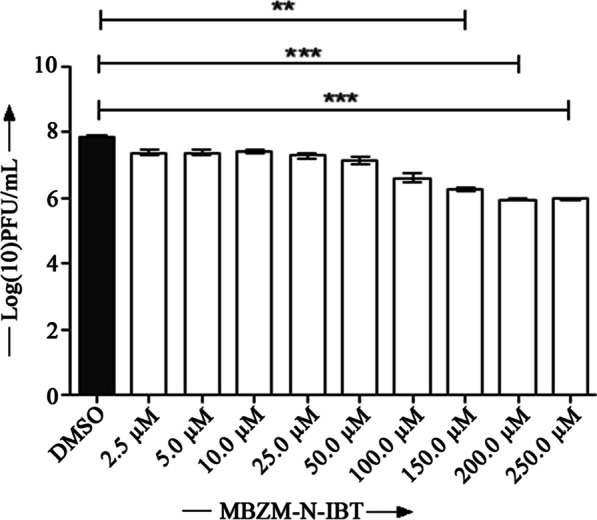
Dose dependent inhibition of HSV-1 using different concentrations of MBZM-N-IBT: Vero cells were infected with HSV-1 and MBZM-N-IBT was added with different concentrations (2.5 µM, 5 µM, 10.0 µM, 25.0 µM, 50.0 µM, 100.0 µM, 150.0 µM and 200.0 µM, 250 µM). DMSO was used as a negative control. The supernatant was collected at 24hpi from the infected and drug treated Vero cells and virus titer was determined by plaque assay. Bar diagram represents the log (10) of PFU/mL of the virus after treatment with different concentrations of MBZM-N-IBT. Data represents as mean ± SEM from three independent experiments using the one way Anova, Dunnett’s multiple comparison test

Next, the IC50 value of MBZM-N-IBT was determined by infecting the Vero cells with HSV-1 and treating with different concentrations (2.5 µM, 5 µM, 10 µM, 50 µM, 100 µM, 150 µM, 200 µM and 250 µM) of MBZM-N-IBT. As shown in Fig. 2 the infectious virus particle number was reduced remarkably after treatment with different concentrations of MBZM-N-IBT. The GraphPad Prism 8 program was used to plot the non-linear regression (dose–response) curve by using the log of concentration against the normalized response. The curve fitting was performed using different options available in the GraphPad Prism 8 program to generate the dose response curve (Additional file 1: Fig 2) and the IC50 value was found to be 3.619 µM.

### MBZM-N-IBT reduces viral DNA and few viral mRNA levels

**Fig. 3:**
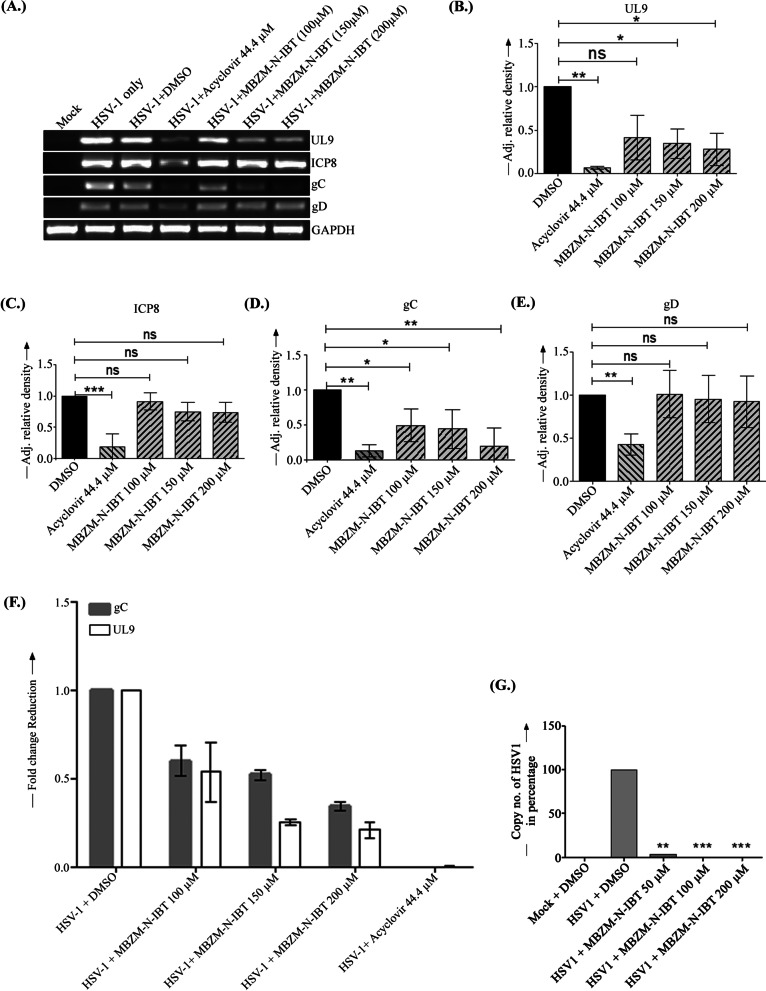
Effect of MBZM-N-IBT in HSV-1 mRNA and DNA levels: **a** Vero cells were infected by HSV-1 with MOI 1 and treated with DMSO (negative control), 44.4 µM Acyclovir (positive control) and MBZM-N-IBT (100 µM, 150 µM, 200 µM,). Total RNA was extracted from the HSV-1 infected cells at 24 hpi, cDNA was synthsized and HSV-1 UL9, ICP8, gC, gD genes were amplified using respective primers by RT-PCR (**a**) Bar diagrams (**b**–**e**) depicts the relative band intensities of viral mRNA expression pattern in infected and drug treated samples of UL9, ICP8, gC and gD genes respectively. Data represent the mean ± SEM from three independent experiments using the one way Anova, Dunnett’s multiple comparison tests.. *p* ≤ 0.05 was considered statistically significant. GAPDH was used as a loading control and the band intensities were normalized based on GAPDH by the ImageJ software. **f** Total RNA was isolated from the HSV-1 infected cells at 24 hpi, cDNA was synthesized and the expression levels of HSV-1 UL9 and gC mRNA, were detected using respective primers by qRT-PCR. Bar diagrams showing the relative fold change reduction in viral mRNA expression pattern in infected and drug treated samples. Data represent the mean ± SEM from three independent experiments using the one way Anova, Dunnett’s multiple comparisons test. *p* ≤ 0.05 was considered statistically significant. GAPDH was used as endogenous control. **g** Vero cells were infected by HSV-1 with MOI 1 and treated with DMSO (negative control) and MBZM-N-IBT (50, 100, 200 µM). Cell supernatants were collected at 24hpi and HSV-1 DNA was isolated by the Qiagen All prep DNA/RNA Mini kit according to the manufacturer’s protocol followed by qPCR of HSV-1 gC gene. Bar diagram represents the viral copy number in percentage which was calculated based on standard curve. Data represent the mean ± SEM from three independent experiments using the one way Anova, Dunnett’s multiple comparisons test

In order to assess the effect in HSV-1 mRNA level, RT-PCR was performed. A significant level of reduction was observed for UL9 (72%) and gC (84%) as compared to the DMSO control (Fig. 3a, b, d). Similar trend was also noticed in quantitative PCR (Fig. 3f). Similar reduction in viral mRNA levels were also demonstrated in the BHK 21 and Raw 267.4 cells (Additional file 1: Fig. 6B & D). However, no significant change was observed for ICP8 and gD in the MBZM-N-IBT treated cells (Fig. 3a, c & e). GAPDH was used as a control. The inhibitory activity of this molecule was also supported by the remarkable reduction of viral copy number as shown in Fig. 3g. This indicates that few mRNA levels and viral DNA were affected by MBZM-N-IBT significantly.

### ICP8 protein level is reduced by MBZM-N-IBT

**Fig. 4:**
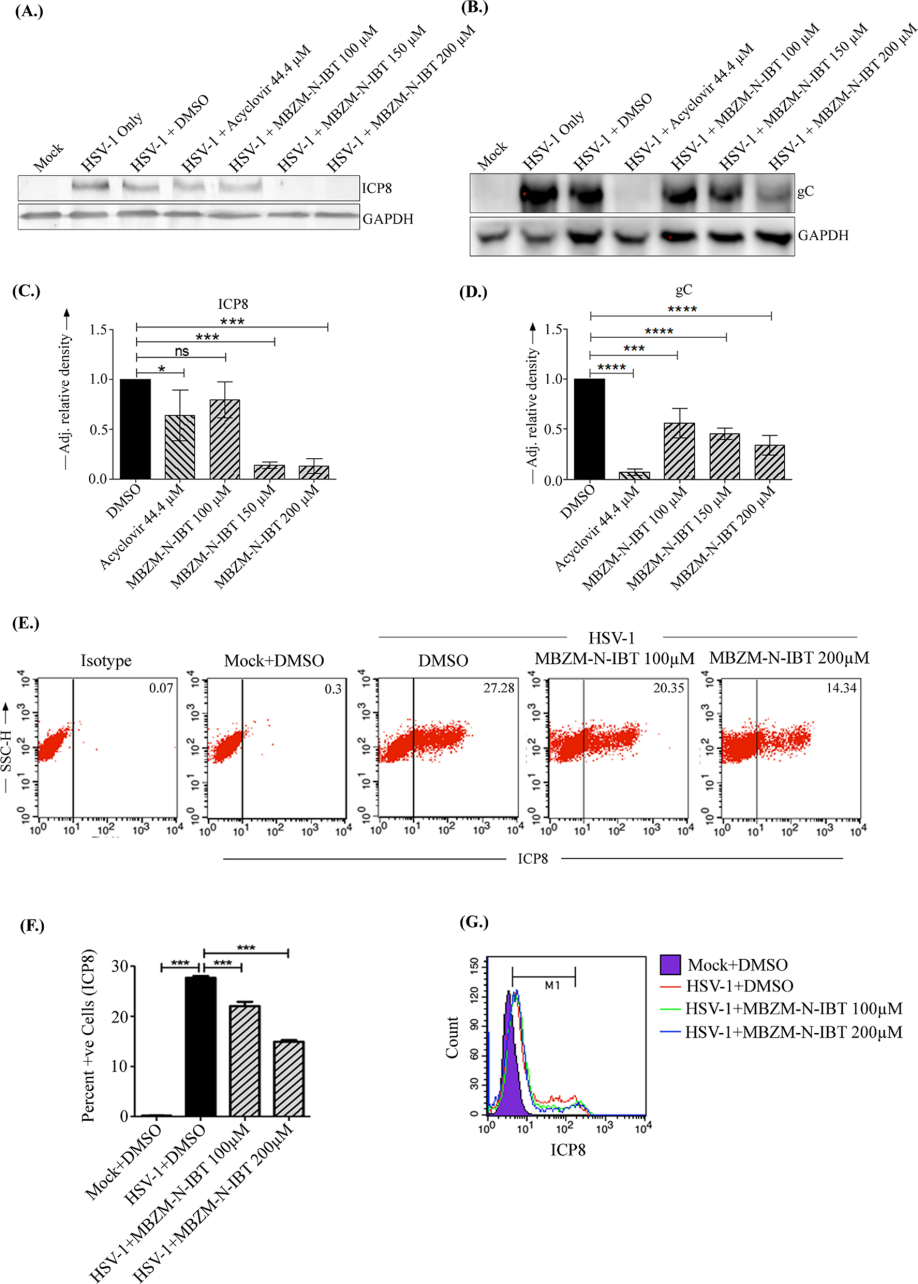
Effect of MBZM-N-IBT in HSV-1 protein levels: Vero cells were infected with HSV-1 at MOI 1 and subsequently treated with DMSO (negative control), Acyclovir (positive control) and different concentration of MBZM-N-IBT (100 µM, 150 µM and 200 μM) and finally harvested at 24 hpi for ICP8 and 15 hpi for gC. The cells were lysed and subjected to Western blot analysis. Western blots were probed with ICP8 **(**a**)** and gC **(b)** antibodies. GAPDH was used as a loading control and the band intensities were normalized based on GAPDH by the ImageJ software. **c, d** Bar diagrams showing the relative band intensities of ICP8 and gC. *p* ≤ *0.05* was considered statistically significant. **e **Flowcytometric dot plot analysis showing percent positive cells for ICP8 at 15 hpi against SSC-H off different samples. **f** Graphical representation showing percent positive cells for ICP8 with varying concentrations of MBZM-N-IBT. **g** Mean fluorescence intensity (MFI) analysis also suggests the ICP8 signal intensity in the presence of MBZM-N-IBT in a dose dependent manner as compared to DMSO control. The comparison between the groups with only one parameter was performed by one way ANOVA (nonparametric) with the Bonferroni post-hoc test*. p* < 0.05 was considered as the statistically significant difference between the groups. (ns, non-significant; ** p ≤ 0.01; *** p ≤ 0.001)

Since MBZM-N-IBT treatment affected levels of few HSV-1 mRNAs, the protein expression levels of ICP8 and gC were studied by Western blot and FACS analysis. Western blot analysis showed 87% reduction in ICP8 and 66% reduction in gC protein levels (Fig. 4a–d). The dot plot analysis of the Flow cytometry data indicated that the percent positive cells for HSV-1 + DMSO were found to be 27.7 ± 0.35 (mean ± SEM), whereas HSV-1 + MBZM-N-IBT was 22.0 ± 0.85 (mean ± SEM) at 100 µM concentration. Moreover, it was reduced to 14.9 ± 0.33 (mean ± SEM) at the 200 µM concentration (Fig. 4e & f). Further, mean fluorescence intensity (MFI) analysis also suggests that there was a reduction in ICP8 in the presence of MBZM-N-IBT in a dose-dependent manner as compared to the DMSO control (Fig. 4g). Taken together, the above data suggest that MBZM-N-IBT reduces HSV-1 specific protein expression without affecting its mRNA level significantly.

### MBZM-N-IBT might interfere in early and late stages of HSV-1 life cycle

**Fig. 5:**
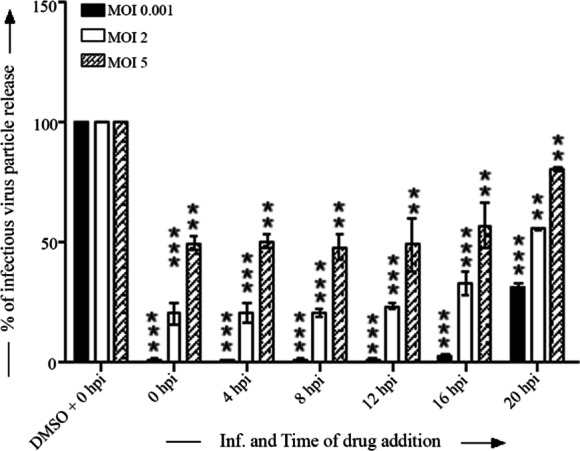
Inhibition pattern of HSV-1 infection by addition of MBZM-N-IBT at different time points: Vero cells were infected by HSV-1 with MOI 0.001, 2, 5 and 200 µM MBZM-N-IBT was added to different dishes at every 4 h intervals up to 20 hpi. The supernatants, as well as cells were collected from all the experimental samples at 20 hpi and plaque assay was performed to assess the number of infectious particles of HSV-1. The bar diagram represents the virus titer in PFU/mL. Data represent the mean ± SEM from three independent experiments using the one way Anova, Dunnett’s multiple comparisons test. *p*-value less than 0.001 was considered to be statistically significant in the test

Time of addition experiment was performed to understand the possible stage of HSV-1 infection that is affected by MBZM-N-IBT. As shown in Fig. 5, it was observed that around 97% of the infectious virus particle release was abrogated after addition of MBZM-N-IBT from 0 to 16 hpi. Even after addition of the drug at 20 hpi, the reduction of the virus particle release was found to be 64% which indicates that a short exposure is sufficient to reduce virus particle release significantly (MOI 0.001). Similar trend was also noticed in case of viral infection with MOI 2 and 5. These findings and the fact that MBZM-N-IBT also significantly inhibits the level of expression of ICP8 protein (Fig. 4a, e–g) suggest that MBZM-N-IBT might interfere in early as well as late stages of the HSV-1 life cycle.

## Discussion

Methisazone, the prototype of thiosemicarbazone antivirals is shown to be effective earlier [14]. However, it was found to be ineffective against CHIKV [13]. Nevertheless, MBZM-N-IBT with similar pharmacophore was reported by us to significantly inhibit CHIKV in vitro by interfering in multiple stages of the CHIKV life cycle [13]. Although the inhibitory activity might not ensure its effectiveness against other viruses, it was thought worthwhile to see if it can also act against herpes. With these objectives, the binding affinities of MBZM-N-IBT against multiple targets of HSV-1 were determined by molecular docking. Since the binding pose was successfully predicted against HSV thymidine kinase (PDB ID: 1KI3), the binding affinity score of this pose was used as the cut-off score. The results revealed higher binding affinities (> 7.3 kcal/Mol) against various targets (Table 1) which suggested its potential for inhibiting HSV. Earlier the host toxicity was found to be low as the CC50 was > 800 µM [13]. Its safety (Category 5: > 500 mg/kg) was also suggested by the oral acute toxicity study carried out as per OCED-423 guidelines (data not shown). Besides, it showed significant anti-inflammatory effects in vivo (data not shown). These are supposed to complement the antiviral effects as inflammation is the key for progress of HSV infection. This encouraged us to verify its inhibitory properties against this virus.

Since CC50 of MBZM-N-IBT was earlier reported to be more than 800 µM, it was screened against HSV-1 at the test concentrations of 100, 150 and 200 µM in the Vero, BHK 21 and Raw 267.4 cell lines. While no toxicity was found at this level, the inhibition was found to be significant in all the cell lines (Fig. 1c, 2, *P* < *0.05*). Following this, screening with different test concentrations (2.5 µM to 250 µM) was conducted and the IC50 value was found to be 3.619 µM (Fig. 2, Additional file 1: Fig. 2). However, further validation in animal model can reveal its suitability for translation to clinical application.

To understand its effectiveness and get an insight into the possible mode of action, the effect of MBZM-N-IBT on the expression of viral mRNA and protein synthesis was investigated. Considering the high number of proteins expressed by HSV, it is difficult to investigate each of them at this stage. Hence, the mRNA expressions of the two non-structural proteins (ICP8 and UL9) and two structural proteins (gC and gD) involved in replication and packaging/egress respectively were studied. While 72% reduction was observed in the mRNA level of UL9, the same for gC was observed to be 84%. Whereas, the reduction observed in the mRNA level of ICP8 and gD were not significant. Considering the high degree of inhibition of mRNA for gC, MBZM-N-IBT was further investigated for its influence on its protein synthesis. Since molecular docking analysis revealed a strong affinity for ICP8 (PDB ID: 1URJ), this was also considered to estimate viral protein synthesis. The results revealed that the protein synthesis of gC and ICP8 were inhibited by 66% and 84% respectively. The relatively lower degree of inhibition of protein synthesis as compared to the mRNA expression of gC suggests that gC might not be a direct target of MBZM-N-IBT. This is also supported by our molecular docking study which shows poor affinity of MBZM-N-IBT against the homologous model of gC protein (Additional file 1: Fig. 3A) developed by our group following established method [18, 21, 22]. In contrast to this, higher binding affinity was predicted for ICP8 (PDB ID: 1URJ) (Additional file 1: Fig. 3B). This was also supported by the in vitro data. A high degree of protein inhibition was observed for ICP8, although the reduction in its mRNA level was not significant. This was further supported by the flow cytometric analysis which showed 47% reduction in ICP8. This suggests that the antiviral effects might have been partly mediated through the inhibition of ICP8. This observation was in agreement with the fact that the viral copy number was also reduced remarkably (Fig. 3). However, further studies are necessary to establish ICP8 as one of the target of MBZM-N-IBT.

Subsequently, the time of addition experiment revealed the ability of MBZM-N-IBT to inhibit HSV-1 at early as well as late stages of infection (Fig. 5). Since, UL9 is the replication initiation protein [23] and ICP8 is the single stranded DNA-binding protein [24] of HSV-1, reduction in mRNA level of UL9 and protein level of ICP8 can partly explain the inhibitory effects of MBZM-N-IBT in early stages. Similarly, a decrease in the expression of mRNA for gC might have contributed to the observed inhibition of HSV-1 at late stages of its life-cycle. Considering the fact that MBZM-N-IBT inhibits early and late stages of HSV-1 infection, the HSV-1 targets cannot be limited to the few mentioned above. It is difficult to experimentally elucidate the interaction of MBZM-N-IBT with all possible targets to justify the inhibition at different stages of the HSV life cycle. Nevertheless, the molecular docking data can be used to predict the possible modes of interference in HSV infection by determining the binding affinities and most stable binding pose with important HSV targets for which valid structures are available. While MBZM-N-IBT showed a poor affinity for HSV-1 thymidine kinase (PDB ID:3FOT), and capsid protein (PDB ID: 1NO7), it showed good affinities for other targets enlisted in Table 1. It showed good interaction (Additional file 1: Fig. 4A) with the C-terminal domain of ICP27 (PDB ID: 5BQK) which is a multifunctional protein conserved across known human HSV-1 [25]. Besides, it showed good interaction (Additional file 1: Fig. 4B) with DNA polymerase UL42 (PDB ID: 2GV9) of HSV-1. In addition to the inhibition of ICP8, this can partly justify the inhibitions observed in the replication stage of HSV-1 life cycle. While affinity for a major capsid protein was poor, interactions with packaging proteins including UL25, DNA packaging protein (PDB ID: 2F5U) and DNA packaging motor pUL15 C-terminal nuclease domain (PDB ID: 4IOX) were good (Additional file 1: Fig. 4C &D). Additionally, a good interaction was also estimated with the extracellular domain of gB (PDB ID: 2GUM) (Additional file 1: Fig. 4E) which is involved in viral attachment and fusion. However, this needs to be experimentally validated. As these proteins are associated with HSV-1 packaging and release [26, 27], their possible inhibition by MBZM-N-IBT can be proposed to be a factor in the justification of the observed inhibition in the late stages of the HSV-1 life cycle. While the binding affinity for HSV-2 protease (PDB ID: 1AT3) was relatively less, it showed higher affinity for the surface envelope glycoprotein D of HSV-2 (Additional file 1: Fig. 5). This suggests that HSV-2 can also be potentially inhibited by MBZM-N-IBT. However, further investigation is necessary to support these suggestions. Also these findings need to be validated in clinical isolates as well as multiple strains of HSV. Nevertheless this finding establishes MBZM-N-IBT as a new hit against HSV. Its effect against CHIKV and HSV makes it an interesting drug candidate for further research to develop new antiviral therapies.

## Conclusion

The emergence of drug resistance to HSV and cross-resistance across nucleoside inhibitors are no longer rare incidences. Antivirals developed during last four decades mostly target DNA polymerases. However, frequent identification of DNA polymerase mutations emphasizes the need to develop antivirals with alternative targets. In this study, MBZM-N-IBT shows anti-HSV action which is possibly mediated through multiple HSV targets including ICP8, ICP27, UL42, UL25, UL15 and gB. Thus, an important future work would be to determine the direct inhibition capacity of MBZM-N-IBT against them. Based on these findings and its good drug-likeness [13], MBZM-N-IBT can be proposed as a new hit for optimization to develop non-nucleoside inhibitors of HSV.

## Supplementary Information

**Additional file 1.Figure 1**: Interaction of penciclovir with HSV thymidine kinase: The ligand and target structures were minimized using the ArgusLab program. Penciclovir was docked against HSV thymidine kinase structure (PDB ID: 1KI3) which was experimentally co-crystallized with penciclovir using the AutoDockVina program. The most stable binding mode visualized by the PyMol software was similar to the experimentally determined mode of interaction.**Figure 2**: Dose response curve of MBZM-N-IBT: Vero cells were infected with HSV-1 and MBZM-N-IBT was added with different concentrations (2.5µM, 5µM, 10.0µM, 25.0µM, 50.0µM, 100.0µM, 150.0µM and 200.0µM, 250 µM). DMSO was used as a negative control. The infected and drug treated cells supernatant were collected after 24 hpi and virus titer was determined by plaque assay. Representation of HSV-1 inhibition curve, where the x-axis depicts the logarithmic value of the concentration of MBZM-N-IBT and y-axis depicted the percent of PFU/mL.**Figure 3**: Interaction of MBZM-N-IBTwith (A) gC and (B) ICP8: The ligand and target structures were minimized using the ArgusLab program . MBZM-N-IBT was docked against homologous model of gC and ICP8 of HSV by the AutoDockVina program. The binding affinities were -6.2Kcal/mole and -9.8Kcal/mole for (A) gC and (B) ICP8. **Figure 4**: Interaction of MBZM-N-IBT with HSV targets: The ligand and target structures were minimized using the ArgusLab program .MBZM-N-IBT was docked against HSV targets involved in multiple stages of its lifecycle using the AutoDockVina program. The binding mode shown by the PyMol software reveals the conformation of the most stable complex with (A) the C-terminal domain of ICP27 protein from HSV-1 (PDB ID : 5BQK), (B) DNA polymerase (UL42) (PDB ID: 2GV9), (C) UL25 DNA packaging protein (PDB ID : 2F5U), (D) DNA-packaging motor pUL15 C-terminal nuclease domain (PDB ID : 4IOX) and (E) extracellular domain of glycoprotein B (PDB ID: 2GUM) respectively. The most stable binding mode of the complex are shown using the PyMol software.**Figure 5**: Interaction of MBZM-N-IBT with HSV-2 surface envelope glycoprotein D The ligand and target structures were minimized using the ArgusLab program MBZM-N-IBT was docked against glycoprotein D (PDB ID: 4MYV) using the AutoDockVina program. The binding mode shown by the PyMol software reveals the conformation of the most stable complex. **Figure 6**: Inhibition of HSV-1 by MBZM-N-IBT in Raw 264.7 and BHK cells: Raw264.7 and BHK cells were infected with HSV-1 (MOI 1) and MBZM-N-IBT was added with different concentrations (50 µM and 100 µM). DMSO was used as a negative control. The infected and drug treated cells and supernatants were collected at 24 hpi and virus titer was determined by plaque assay. A and C. represents the percent of PFU/mL of the virus after treatment with different concentrations of MBZM-N-IBT in Raw 264.7 cells and BHK cells respectively. B, D depicts the fold changes of gC and UL9 genes in their RNA levels in HSV-1 infected Raw264.7 and BHK cells respectively. Data represent the mean ± SEM from three independent experiments using the one way Anova, Dunnett’s multiple comparison tests.. p≤ 0.05 was considered be to statistically significant.**Table 1** Primers used in RT-PCR

## Data Availability

Not applicable.

## Abbreviations

MBZM-N-IBT: 1-[(2-Methyl benzimidazole-1-yl) Methyl]-2-Oxo-Indolin-3-ylidene] Amino] Thiourea
HSV: Herpes simplex viruses
MOI: Multiplicity of infection
PBS: Phosphate-buffered saline
DMEM: Modified Eagle’s Medium
FBS: Fetal bovine serum
PFU: Plaque Forming Unit
CPE: Cytopathic effect
