# Supplementary material for: Inhibition of herpes simplex virus-1 infection by MBZM-N-IBT: in silico and in vitro studies
Source: Virol J. 2021 May 26;18:103. doi: 10.1186/s12985-021-01581-5 (PMC8157732; doi:10.1186/s12985-021-01581-5)
Supplement: Supplementary file 1 — Additional file 1.Figure 1: Interaction of penciclovir with HSV thymidine kinase: The ligand and target structures were minimized using the ArgusLab program. Penciclovir was docked against HSV thymidine kinase structure (PDB ID: 1KI3) which was experimentally co-crystallized with penciclovir using the AutoDockVina program. The most stable binding mode visualized by the PyMol software was similar to the experimentally determined mode of interaction.Figure 2: Dose response curve of MBZM-N-IBT: Vero cells were infected with HSV-1 and MBZM-N-IBT was added with different concentrations (2.5µM, 5µM, 10.0µM, 25.0µM, 50.0µM, 100.0µM, 150.0µM and 200.0µM, 250 µM). DMSO was used as a negative control. The infected and drug treated cells supernatant were collected after 24 hpi and virus titer was determined by plaque assay. Representation of HSV-1 inhibition curve, where the x-axis depicts the logarithmic value of the concentration of MBZM-N-IBT and y-axis depicted the percent of PFU/mL.Figure 3: Interaction of MBZM-N-IBTwith (A) gC and (B) ICP8: The ligand and target structures were minimized using the ArgusLab program . MBZM-N-IBT was docked against homologous model of gC and ICP8 of HSV by the AutoDockVina program. The binding affinities were -6.2Kcal/mole and -9.8Kcal/mole for (A) gC and (B) ICP8. Figure 4: Interaction of MBZM-N-IBT with HSV targets: The ligand and target structures were minimized using the ArgusLab program .MBZM-N-IBT was docked against HSV targets involved in multiple stages of its lifecycle using the AutoDockVina program. The binding mode shown by the PyMol software reveals the conformation of the most stable complex with (A) the C-terminal domain of ICP27 protein from HSV-1 (PDB ID : 5BQK), (B) DNA polymerase (UL42) (PDB ID: 2GV9), (C) UL25 DNA packaging protein (PDB ID : 2F5U), (D) DNA-packaging motor pUL15 C-terminal nuclease domain (PDB ID : 4IOX) and (E) extracellular domain of glycoprotein B (PDB ID: 2GUM) respectively. The most stable binding mod [file 12985_2021_1581_MOESM1_ESM.docx]

**Inhibition of Herpes Simplex Virus-1 infection by MBZM-N-IBT: *In silico* and *in vitro* studies**

Abhishek Kumar^1^, Saikat De^1^, Alok Kumar Moharana^2^, Tapas Kumar Nayak^1^, Tanuja Kumari^1^, Ankita Datey^1^, Prabhudutta Mamidi^1^, Priyadarshee Mishra^2^, Bharat Bhusan Subudhi^2*^ and Soma Chattopadhyay^1*^

^1^Institute of Life Sciences, Bhubaneswar, India

^2^School of Pharmaceutical Sciences, Siksha O Anusandhan Deemed to be University, Bhubaneswar, India

*Address of Corresponding authors:

Soma Chattopadhyay

Institute of Life Sciences,

Autonomous Institute of Dept of Biotechnology (Govt of India),

Nalco Square, Bhubaneswar-751023, India

Phone No: 0091 674 2304235; Fax No: 0091 674 2300728

Email: sochat.ils@gmail.com

and

Bharat Bhusan Subudhi

School of Pharmaceutical Sciences, Siksha O Anusandhan Deemed to be University

Khandagiri Square, Bhubaneswar-751003, India

Phone No: 09853945363; Email: bharatbhusans@gmail.com


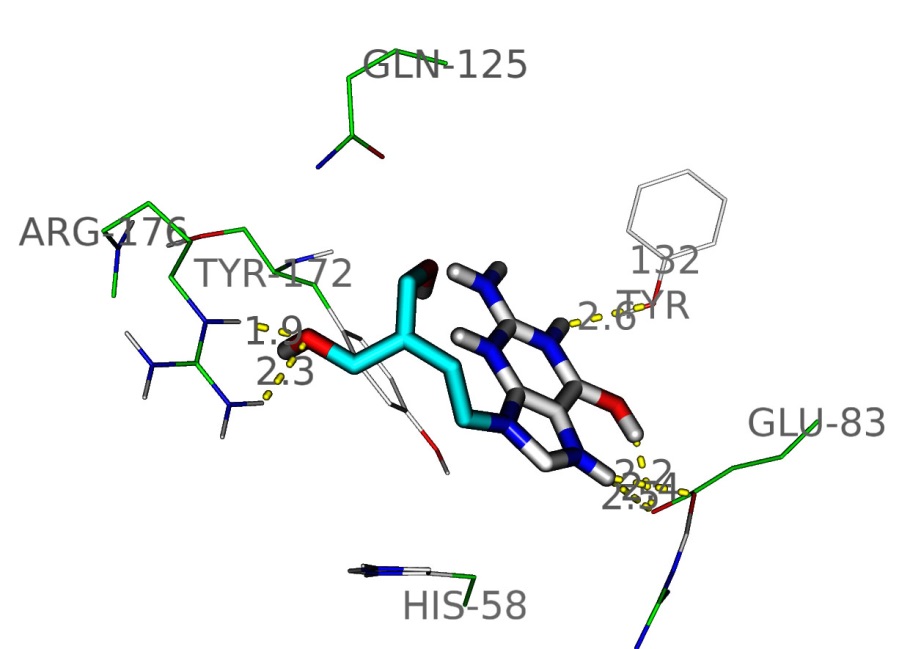


**Additional File 1. Figure 1: Interaction of penciclovir with HSV thymidine kinase:** Penciclovir was docked against HSV thymidine kinase structure (PDB ID: 1KI3) which was experimentally co-crystallized with penciclovir using the AutoDock Vina program. The most stable binding mode visualized by the PyMol software was similar to the experimentally determined mode of interaction.


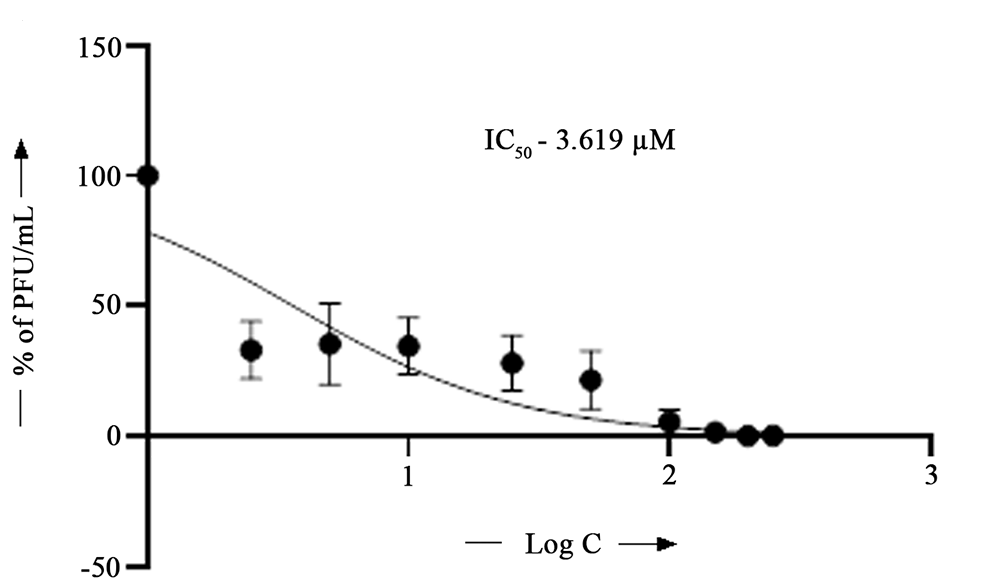


**Additional File 1. Figure 2: Dose response curve of MBZM-N-IBT**: Vero cells were infected with HSV-1 and MBZM-N-IBT was added with different concentrations (2.5µM, 5µM, 10.0µM, 25.0µM, 50.0µM, 100.0µM, 150.0µM and 200.0µM, 250 µM). DMSO was used as a negative control. The infected and drug treated Vero cells and supernatants were collected after 24 hpi and virus titer was determined by plaque assay. Representation of HSV-1 inhibition curve, where the x-axis depicts the logarithmic value of the concentration of MBZM-N-IBT and y-axis depicted the percent of PFU/ML

| **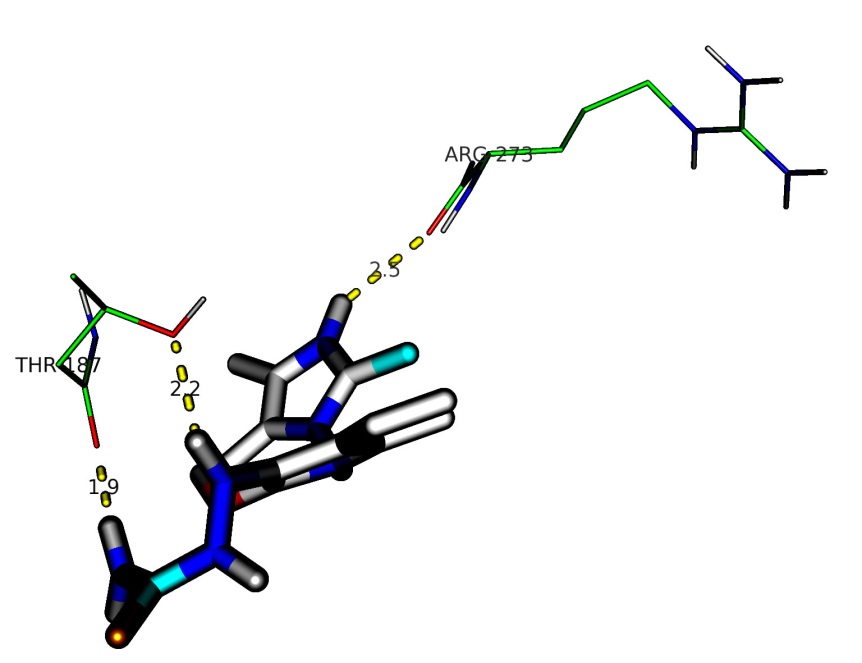** |
| --- |
| **(A)** |
| 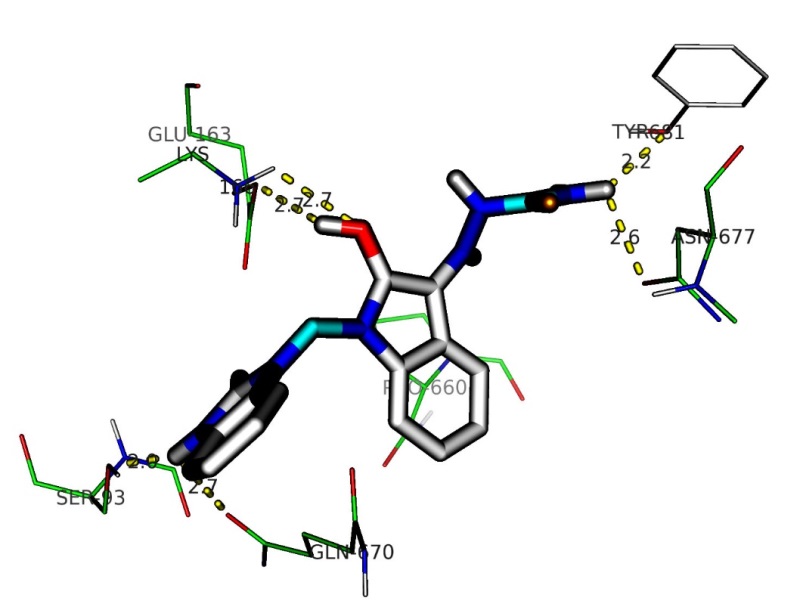 |
| **(B)** |

**Additional File 1. Figure 3: Interaction of MBZM-N-IBT** **with (A) gC and (B) ICP8:** MBZM-N-IBT was docked against homologous model of gC and ICP8 of HSV by the AutoDock Vina program. The binding affinities were -6.2Kcal/mole and -9.8Kcal/mole for (A) gC and (B) ICP8 respectively. The most stable binding mode of the complex are shown using the PyMol software.

**Additional File 1. Figure 4:**
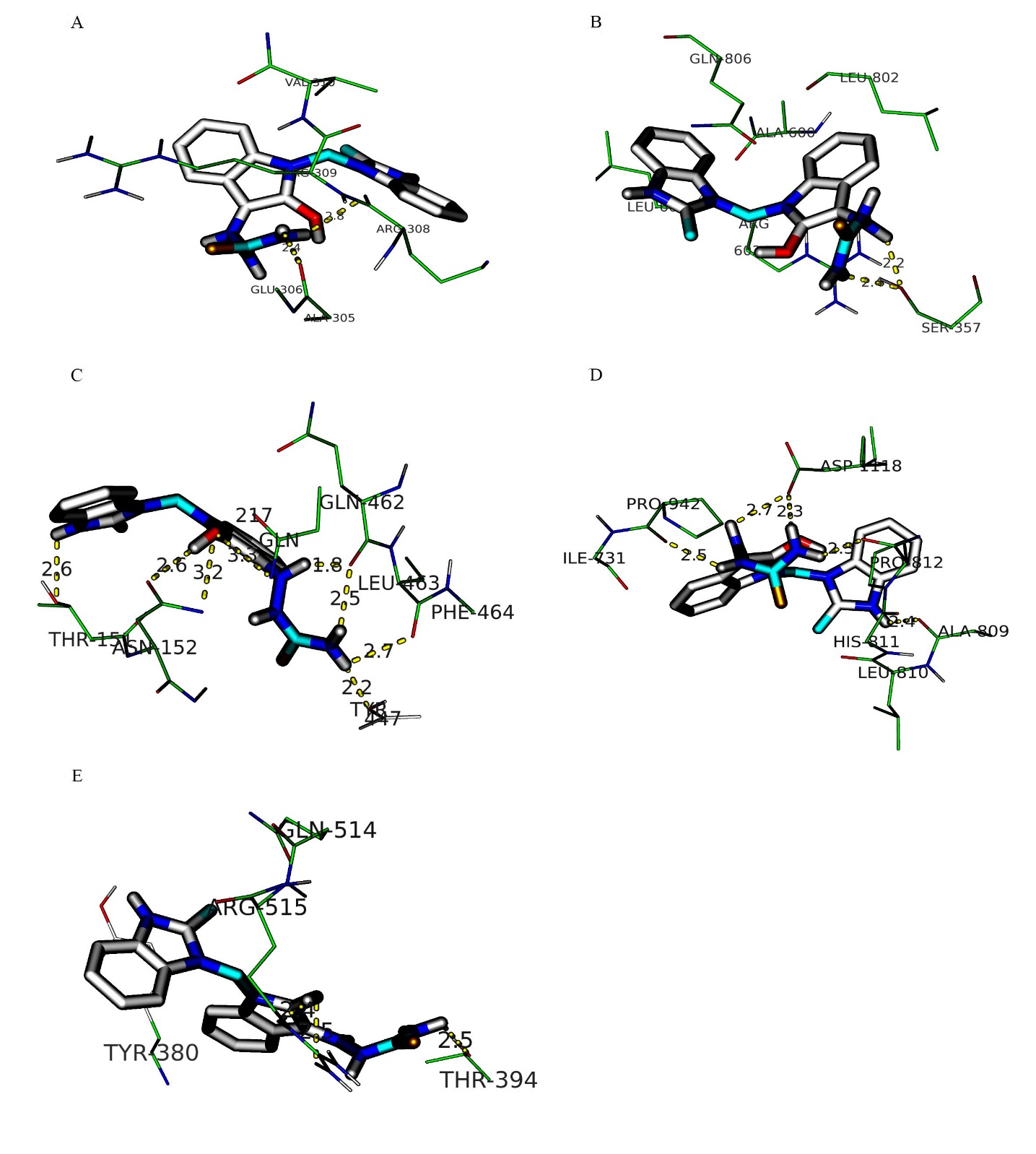
**Interaction of MBZM-N-IBT with HSV targets:** MBZM-N-IBT was docked against HSV targets involved in multiple stages of its lifecycle using the AutoDock Vina program. The binding mode shown by the PyMol software reveals the conformation of the most stable complex with **(A)** the C-terminal domain of ICP27 protein from HSV-1 (PDB ID : 5BQK), **(B)** DNA polymerase (UL42) (PDB ID: 2GV9), **(C)** UL25 DNA packaging protein (PDB ID : 2F5U), **(D)** DNA-packaging motor pUL15 C-terminal nuclease domain (PDB ID : 4IOX) and **(E)** extracellular domain of glycoprotein B (PDB ID: 2GUM).


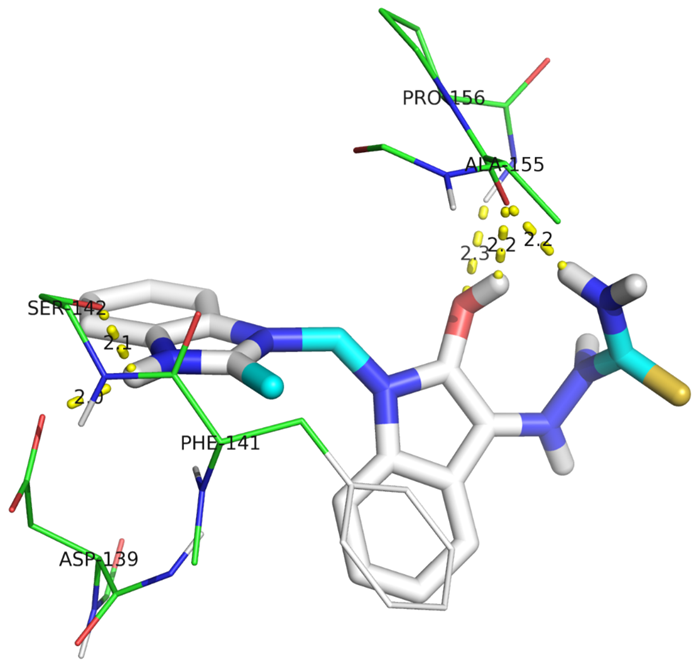


**Additional file 1. Figure 5: Interaction of MBZM-N-IBT with HSV-2 surface envelope glycoprotein D:** MBZM-N-IBT was docked against glycoprotein D (PDB ID: 4MYV) using the AutoDock Vina program. The binding mode shown by the PyMol software reveals the conformation of the most stable complex**.**


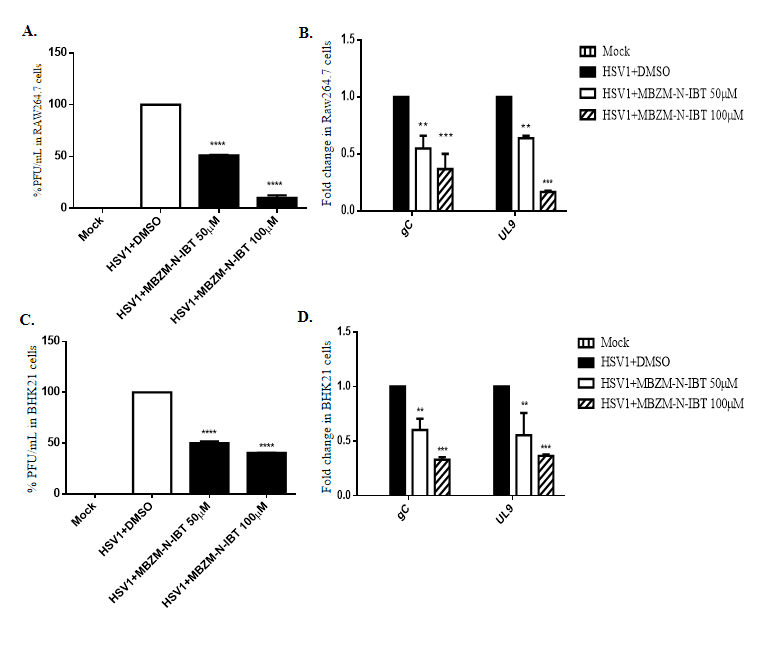


**Additional file 1. Figure 6: Inhibition of HSV-1 by MBZM-N-IBT in Raw 264.7 and BHK cells:** Raw264.7 and BHK cells were infected with HSV-1 (MOI 1) and MBZM-N-IBT was added with different concentrations (50 µM and 100 µM). DMSO was used as a negative control. The infected and drug treated cells and supernatants were collected at 24 hpi and virus titer was determined by plaque assay. A and C. represents the percent of PFU/mL of the virus after treatment with different concentrations of MBZM-N-IBT in Raw 264.7 cells and BHK cells respectively. B, D depicts the fold changes of gC and UL9 genes in their RNA levels in HSV-1 infected Raw264.7 and BHK cells respectively. Data represent the mean ± SEM from three independent experiments using the one way Anova, Dunnett’s multiple comparison tests.. p≤ 0.05 was considered be to statistically significant.

| Sl. No. | Gene | Primer Name | Sequence | Product size |
| --- | --- | --- | --- | --- |
| 1 | UL9 | HUL9F | 5′CCGCGTCCAACCGTTTATTA3′ | 207bp |
| 2 |  | HUL9R | 5'GCGACAACACTGACCATCTT 3′ |  |
| 3 | gD | HgDF | 5′GTCCGGAAACAACCCTACAA3′ | 264bp |
| 4 |  | HgDR | 5′CTCCGTCCAGTCGTTTATCTTC3′ |  |
| 5 | ICP8 | HICP8F | 5′GGGTGTAGTCCGAAAAGCCA3′ | 338bp |
| 6 |  | HICP8R | 5′CCTGGGATACGTGTACGCTC3′ |  |
| 7 | gC | HgCF | 5′TGATTATCGGCGAGGTGACG3′ | 214bp |
| 8 |  | HgCR | 5′CAAACTCCACGGGGTTACG3′ |  |
| 9 | GAPDH | GAPDHF | 5′CAAGGTCATCCATGACAACTTTG3′ | 500bp |
| 10 |  | GAPDHR | 5′GTCCACCACCCTGTTGCTGTAG3′ |  |

**Supplemental Table 1: Primers used in RT-PCR for different genes of HSV-1.**
